# Clinical Effects of the Neutrophil-to-Lymphocyte Ratio/Serum Albumin Ratio in Patients with Gastric Cancer after Gastrectomy

**DOI:** 10.3390/jpm13030432

**Published:** 2023-02-28

**Authors:** Shizune Onuma, Itaru Hashimoto, Hideaki Suematsu, Shinsuke Nagasawa, Kyohei Kanematsu, Toru Aoyama, Takanobu Yamada, Yasushi Rino, Takashi Ogata, Takashi Oshima

**Affiliations:** 1 Department of Gastrointestinal Surgery, Kanagawa Cancer Center, Yokohama 241-8515, Japan; 2 Department of Surgery, Yokohama City University, Yokohama 236-0004, Japan

**Keywords:** gastric cancer, prognostic factor, neutrophil-to-lymphocyte ratio/albumin ratio

## Abstract

Preoperative inflammatory and nutritional statuses have potential prognostic effects on patients with gastric cancer (GC) after curative gastrectomy. We investigated the prognostic usefulness of the preoperative neutrophil-to-lymphocyte ratio/albumin ratio (NLR/Alb) in patients with GC. Among 483 patients who underwent gastrectomy for GC, the preoperative prognostic nutritional index (PNI), NLR, and NLR/Alb were calculated using preoperative blood test data. The patients were divided into the high and low PNI, NLR, and NLR/Alb groups. The associations of preoperative PNI, NLR, and NLR/Alb with clinicopathological features, 3-year (3Y) overall survival (OS) rates, and relapse-free survival (RFS) rates after gastrectomy for GC were evaluated. The number of female individuals and the C-reactive protein levels were significantly higher in the high- compared to the low-NLR/Alb group (both *p* < 0.05). The 3Y OS and 3Y RFS rates following gastrectomy were significantly lower in the high- compared to the low-NLR/Alb group (88.2% vs. 97.8%, *p* = 0.003 and 84.2% vs. 95.6%, *p* = 0.002, respectively). In multivariate analysis, high NLR/Alb could independently predict prognosis and recurrence (hazard ratio [HR]: 4.13; 95% confidence interval [CI]: 1.26–13.55; *p* = 0.02 and HR: 3.16; 95% CI: 1.34–7.45, *p* = 0.009, respectively). Preoperative NLR/Alb might be a useful prognostic factor for patients with GC after curative gastrectomy.

## 1. Introduction

Gastric cancer (GC) is the fifth most common cause of new cancers and the third leading cause of cancer-related deaths [1]. In general, the standard treatment for clinical early-stage or resectable advanced GC is endoscopic resection or laparoscopic or open gastrectomy with lymph node dissection [2]. For adjuvant chemotherapy after surgical treatment, S-1 monotherapy is recommended according to the Adjuvant Chemotherapy Trial of S-1 for Gastric Cancer (ACTS-GC) [3]. Moreover, S-1 plus docetaxel is recommended for pathologic (p)Stage III GC cases based on the results of the JACCRO GC-07 trial, which showed that the treatment combination improved survival in pStage III GC [4]. Recently, novel systemic chemotherapy regimens for metastatic gastric cancer/gastroesophageal junction cancer have become available, including nivolumab [5,6]; pembrolizumab [7], based on microsatellite instability status; trastuzumab deruxtecan, based on human epidermal growth factor receptor type 2 status [8]; and trifluridine/tipiracil [9]. Despite recent advancements in diagnosis, surgery, and systemic chemotherapy with clinical parameters, the outcomes of GC treatments remain poor and can be improved [2]. Therefore, the search for prognostic factors for patients with GC is of great importance. Recently, reports on the search for novel prognostic factors and risk assessment scores of surgical complications using methods such as machine learning based on artificial intelligence, as well as peripheral blood and clinicopathological data derived from patients, have gained attention [10,11].

To date, the preoperative immune status and nutritional status, which can be evaluated by the neutrophil-to-lymphocyte ratio (NLR) and the prognostic nutritional index (PNI = 10 × serum albumin [Alb] [g/dL] + 0.005 × total lymphocyte count [/μL]), in cancer treatment outcomes have been reported and have attracted considerable attention. Several studies have reported that preoperative NLR and PNI are prognostic factors in patients with GC after surgery [12,13,14,15,16,17].

Furthermore, the search for novel indices that accurately reflect the nutritional and immunological status of patients with cancer has been pursued from a prognostic viewpoint. NLR/Alb, as a combined immune status and nutritional index, was first reported by Zhao et al. [18] to minimize the potential bias related to the nutritional and immune statuses of patients with cancer and has been a novel and strong prognostic factor for several cancers, including esophageal squamous cell carcinoma (ESCC) [18,19] and renal cell cancer (RCC) [20]. However, there are no reports on preoperative NLR/Alb as a prognostic factor for GC after gastrectomy. Hence, this study aimed to evaluate preoperative NLR/Alb using preoperative peripheral blood tests and to clarify its prognostic effects for patients with GC who undergo curative surgery.

## 2. Materials and Methods

### 2.1. Patients

**Figure 1:**
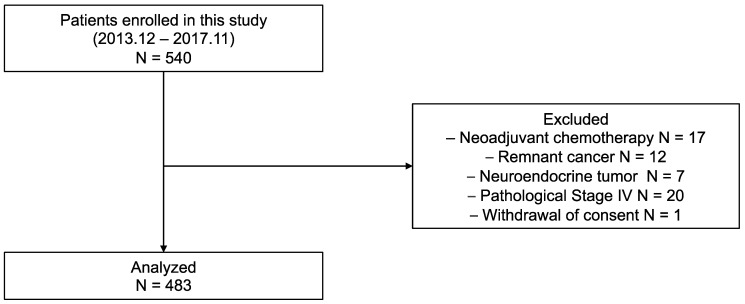
Flowchart of the patient selection process.

Between December 2013 and November 2017, 540 patients with GC were enrolled in this study. The registration criteria were as follows: (i) GC confirmed by pathological diagnosis, (ii) gastrectomy achieving R0 resection with radical lymph node resection as the initial treatment for GC, (iii) age > 20 years, and (iv) Eastern Cooperative Oncology Group performance status score of 0–2 points. Fifty-seven patients who received neoadjuvant chemotherapy, had remnant cancer, had a neuroendocrine tumor, had a diagnosis of pStage IV, or who withdrew their consent were excluded from this study (Figure 1).

In principle, patients with pathological stage (pStage) II disease received S-1 monotherapy, and patients with pStage III disease received S-1 plus docetaxel or capecitabine plus oxaliplatin therapy for 1 year. All study protocols were approved by the Ethics Committee of Kanagawa Cancer Center (approval number: 25Research-20), and all procedures were conducted in accordance with the Declaration of Helsinki of 1996.

### 2.2. Measurement of the PNI, NLR, and Alb

We calculated PNI, NLR, and NLR/Alb levels, using preoperative blood test data, as follows:

PNI = 10 × Alb (g/dL) + 0.005 × lymphocytes (/μL)

NLR = neutrophil (/μL)/lymphocyte (/μL)

NLR/Alb = neutrophil (/μL)/lymphocyte (/μL)/Alb (g/dL).

The cutoff values, defined using a receiver operating characteristic curve analysis [21,22] for survival and death, were as follows: PNI (48.3), NLR (1.64), and NLR/Alb (0.39) (Figure S1). The patients were divided into the high and low PNI, NLR, and NLR/Alb groups.

### 2.3. Analyzed Parameters

The 3-year overall survival (3Y OS) and 3-year relapse-free survival (3Y RFS) rates after gastrectomy for GC were calculated for each clinicopathological parameter. Prognostic factors were analyzed using the following variables: patient age, sex, body mass index (BMI), preoperative C-reactive protein (CRP) level, surgery, tumor size, histological type, lymphatic invasion, venous invasion, pStage, postoperative complications, and hospitalization days.

### 2.4. Statistical Analyses

Continuous variables were presented as means ± standard deviations and were nonparametrically analyzed using the Mann–Whitney U test after the Kolmogorov–Smirnov test. Categorical variables were compared using a χ^2^ or Fisher’s exact test, as appropriate. The 3Y OS and 3Y RFS rates were evaluated using the Kaplan–Meier method and log-rank test, respectively. Variables identified as significant (*p* < 0.05) in univariate analysis were considered candidates for multivariate Cox regression analysis, and the results are shown as hazard ratios (HRs) with 95% confidence intervals (CIs). Statistical significance was set at *p* < 0.05. All statistical analyses were performed using EZR (Saitama Medical Center, Jichi Medical University, Saitama, Japan), a graphical user interface for R (The R Foundation for Statistical Computing, Vienna, Austria).

## 3. Results

### 3.1. Association of PNI, NLR, and NLR/Alb with Clinicopathological Factors

Table 1 and Table S1 show the association of the preoperative PNI, NLR, and NLR/Alb with clinicopathological factors in patients with GC.

Overall, 483 patients were categorized into one of two groups according to their PNI, NLR, and NLR/Alb as follows: high PNI (*n* = 292) or low PNI (*n* = 191), high NLR (*n* = 339) or low NLR (*n* = 147), and high NLR/Alb (*n* = 344) or low NLR/Alb (*n* = 139) (Table 1 and Table S1). The patients in the low-PNI group were older (*p* < 0.001), were predominantly female (*p* = 0.02), had lower BMI values (*p* < 0.001), and had higher preoperative CRP levels (*p* < 0.001) than those in the low-NLR group. Moreover, the number of total gastrectomy performed was higher (*p* = 0.01), the tumor size was larger (*p* < 0.001), there were more lymphatic invasions (*p* = 0.01) and venous invasions (*p* = 0.003), and there were more cases of pStage II/III disease (*p* < 0.001) in the low- compared to the high-PNI group. The patients in the high-NLR group had higher preoperative CRP levels (*p* = 0.02) than those in the low-NLR group. The patients in the high-NLR/Alb group were predominantly female (*p* < 0.05) and had higher C-reactive protein levels (*p* < 0.05) than those in the low-NLR/Alb group.

### 3.2. 3Y OS and RFS Rates According to PNI, NLR, and NLR/Alb

**Figure 2:**
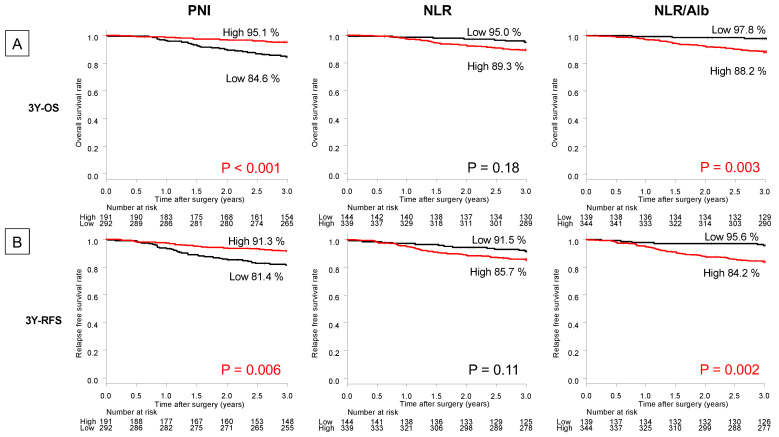
Kaplan–Meier 3-year (**A**) overall survival (OS) and (**B**) relapse-free survival (RFS) rates according to the prognostic nutritional index (PNI), neutrophil-to-lymphocyte ratio (NLR), and NLR/albumin (Alb). The red words indicate statistically significant (*p* < 0.05).

The 3Y OS rates following gastrectomy in patients with high and low PNI were 95.1% and 84.6%, respectively (*p* < 0.001) (Figure 2A). The 3Y OS rates following gastrectomy in patients with high and low NLR were 89.3% and 95.0%, respectively (*p* = 0.18) (Figure 2A). Moreover, the 3Y OS rates following gastrectomy in patients with high NLR/Alb and low NLR/Alb were 88.2% and 97.8%, respectively (*p* = 0.003) (Figure 2A).

The 3Y RFS rates following gastrectomy in patients with high and low PNI were 81.4% and 91.3%, respectively (*p =* 0.006) (Figure 2B).

The 3Y RFS rates following gastrectomy in patients with high and low NLR were 85.7% and 91.5%, respectively (*p* = 0.11) (Figure 2B). The 3Y RFS rates following gastrectomy in patients with high NLR/Alb and low NLR/Alb were 84.2% and 95.6%, respectively (*p* = 0.002) (Figure 2B).

Survival analysis based on TNM classification revealed that the patients with high NLR/Alb had significantly poorer prognoses for 3Y OS and 3Y RFS rates than those with low NLR/Alb, especially those with pStage II/III GC (74.3% vs. 94.5%, *p* = 0.005 and 63.7% vs. 92.0%, *p* = 0.002) (Figure S2). Meanwhile, those with high NLR/Alb did not have significantly poorer prognoses for the 3Y OS and 3Y RFS rates compared to those with low NLR/Alb in pStage I GC (95.2% vs. 99.0%, *p* = 0.40 and 94.3% vs. 97.0%, *p* = 0.69) (Figure S2).

### 3.3. Univariate and Multivariate Analyses

Table 2 and Table 3 show the results of the univariate and multivariate analyses of 3Y OS and 3Y RFS rates among patients with GC who underwent gastrectomy according to the NLR/Alb.

In the multivariate analyses, venous invasion (HR: 3.81; 95% CI: 1.50–9.68; *p* = 0.005), pStage (HR: 2.65; 95% CI: 1.21–5.81; *p* = 0.02), and NLR/Alb (HR: 4.13; 95% CI: 1.26–13.55; *p* = 0.02) were independent prognostic factors for 3Y OS (Table 2). Moreover, in multivariate analyses, venous invasion (HR: 2.13; 95% CI: 1.03–4.42; *p* = 0.04), pStage (HR: 3.48; 95% CI: 1.68–7.20; *p* < 0.001), and NLR/Alb (HR: 3.16; 95% CI: 1.34–7.45; *p* = 0.009) were independent prognostic factors for 3Y RFS (Table 3). Tables S2 and S3 show the results of the univariate and multivariate analyses of the 3Y OS and 3Y RFS rates among patients with GC who underwent gastrectomy according to PNI. In the multivariate analyses, venous invasion (HR: 3.67; 95% CI: 1.44–9.36; *p* = 0.007) and pStage (HR: 2.35; 95% CI: 1.05–5.28; *p* = 0.04) were independent prognostic factors for 3Y OS (Table S2). Nevertheless, PNI was not an independent prognostic factor (HR: 1.82; 95% CI: 0.92–3.57; *p* = 0.09). Lymphatic invasion (HR: 1.86; 95% CI: 1.02–3.39; *p* = 0.04), venous invasion (HR: 2.13; 95% CI: 1.03–4.41; *p* = 0.04), and pStage (HR: 3.24; 95% CI: 1.56–6.71; *p* = 0.002) were also independent prognostic factors for 3Y RFS in multivariate analyses (Table S3). However, PNI was not an independent prognostic factor (HR: 1.37; 95% CI: 0.79–2.38; *p* = 0.26).

## 4. Discussion

Peri-treatment immune and nutritional status has attracted scientific attention as a prognostic factor for patients with cancer [23,24]. NLR, as an immune status index, and PNI, as a nutritional status index, have been useful prognostic factors in patients with GC undergoing surgery [20,25,26]. However, the combined immune status and nutritional assessment indices have not been adequately evaluated. Hence, in this study, we focused on the NLR, PNI, and NLR/Alb, as a combined index, to investigate their usefulness as novel prognostic factors for patients with GC after surgery. In survival analyses, the 3Y OS and 3Y RFS in the low-PNI and high-NLR/Alb groups were significantly lower than those in the high-PNI and low-NLR/Alb groups. In addition, low PNI and high NLR/Alb levels were significant prognostic factors for 3Y OS. Furthermore, high NLR/Alb was a significant prognostic factor for 3Y RFS, whereas low PNI values were not a prognostic factor for 3Y RFS. Hence, NLR/Alb, as a combined immune status and nutritional index, provided more insight into prognostic predictions for patients with GC after gastrectomy.

To date, novel indices focusing on immune status and nutritional status have been reported as prognostic factors for patients with GC. A high NLR, reflecting immune status, is associated with poor OS [27], disease-specific survival [28], and disease-free survival (DFS) [29] of patients with GC after surgery. In addition, a low PNI, reflecting malnutrition, was associated with poor OS [26,30] and DFS [17] in patients with GC after surgery. Some studies have shown the usefulness of the combined immune status and nutritional assessment indices, such as the NLR/Alb, NLR/pre-Alb, and CRP/Alb ratios, in several cancers. A retrospective study reported that patients with high NLR/Alb had significantly worse cancer-specific survival (CSS) than those with low NLR/Alb (11.0% vs. 39.1%, *p* < 0.001) in ESCC [18]. In multivariate analysis, a high NLR/Alb level was an independent prognostic factor for CSS (*p* = 0.001). In addition, a retrospective study showed that patients with ESCC with high NLR/pre-Alb had worse OS than those with low NLR/pre-Alb (*p* = 0.043) [19]. According to a previous multivariate analysis, a high NLR/pre-Alb was an independent prognostic factor for OS (*p* = 0.045). Furthermore, a retrospective study showed that those with high NLR/Alb and CRP/Alb ratios had significantly worse PFS and OS than those with low NLR/Alb and CRP/Alb ratios in advanced RCC cases [20]. According to a previous multivariate analysis, high NLR/Alb and CRP/Alb ratios were independent prognostic factors for PFS and OS, respectively. Although no reports exist on the use of NLR/Alb as a prognostic marker in GC, several reports exist on other combined inflammatory and nutritional assessment indices, such as CAR [31], CRP/serum pre-albumin [32], the lymphocyte-to-CRP ratio [33], and the Naples prognostic score (NPS) [34]. In comparison to these combined indices that include two components (serum protein and lymphocyte), the NLR/Alb, which contains three components (serum albumin, neutrophils, and lymphocytes), may better reflect cancer patients’ immune and nutritional statuses; the results of our analyses support this hypothesis. In contrast, the NLR/Alb index is more straightforward compared to the NPS, which is calculated using the total cholesterol level, albumin, NLR, and lymphocyte-to-monocyte ratio. Therefore, NLR/Alb can help predict the prognosis of patients with GC after gastrectomy.

Low immune status and malnutrition play a crucial role in cancer progression and prognosis [35,36,37]. Although preoperative NLR/Alb can be a useful prognostic factor for survival after surgery, its detailed mechanism remains unclear. We propose several hypotheses to explain the mechanism of NLR/Alb as a useful prognostic marker in patients with GC. A high NLR reflects an increased neutrophil response and a decreased lymphocyte response to the tumor. Neutrophils play an important role in cancer progression, angiogenesis, epithelial–mesenchymal transition (EMT), and immunosuppression [38,39]. In GC cell line analysis, neutrophils at invasive lesions regulate tumor angiogenesis via matrix metalloproteinase-9 [40]. Furthermore, neutrophils accelerate GC cell invasion and migration through the ERK pathway and EMT via the pro-inflammatory factors interleukin (IL)-1β, IL-6, IL-8, and tumor necrosis factor-α [41]. From an immunological perspective, activated programmed death ligand-1-positive neutrophils in GC tissue suppress T-cell function, inducing tumor progression [42]. In vitro co-culture experiments and immunohistochemical staining have shown that tumor-associated neutrophils decreased the proliferation of CD4+ T cells and upregulated the expression of programmed cell death protein 1, which might promote an immunosuppressive cancer environment in GC [43]. Lymphocytes, particularly CD8+ T cells [44,45] and memory T cells [46], play a crucial role in antitumor immune function and inhibit the progression of several cancers. Peripheral blood analysis in patients with GC revealed that toll-like receptor 2 expression in CD8+ T cells was downregulated, which might be associated with immune dysfunction through the suppression of the perforin–granzyme pathway [47]. In stage II/III GC, blood test analyses of 3243 patients showed that a low lymphocyte count was an independent prognostic factor [48]. In addition, serum albumin level reflects inflammation and malnutrition in cancer hosts [49]. A retrospective study of 1023 patients with GC showed that pre-therapeutic serum albumin level was a significant prognostic factor [50]. Furthermore, our prospective study of 500 patients with GC demonstrated that preoperative NLR/Alb is a prognostic factor for survival after curative surgery. Thus, preoperative NLR/Alb, which is a combination index using both the immune and nutritional status, may be a useful prognostic index in GC after curative surgery.

This study showed that patients with high NLR/Alb tended to have a poor prognosis, whereas those with low NLR/Alb had a good prognosis after gastrectomy, especially for advanced GC. Regarding clinical application, the preoperative NLR/Alb may be useful as a powerful prognostic predictor. An assessment of immune and nutritional status is important in predicting the prognosis of patients with GC undergoing resection of the stomach, which is essential for digestion and absorption. Thus, preoperative NLR/Alb may help select patients for aggressive perioperative nutritional intervention, which may contribute to fewer complications and better outcomes by improving nutritional status [51]. In addition, it can guide the selection of patients with GC who should receive neoadjuvant chemotherapy [52,53]. Furthermore, it may be used to select a more appropriate treatment strategy for patients with advanced GC who are indicated for adjuvant chemotherapy (stage II: S-1 monotherapy, stage III: S-1 plus docetaxel) [3,4], supported by pStage diagnosis.

This study has some limitations. First, as this was a single cohort and there are few reports on NLR/Alb to date, a clinically relevant cutoff of NLR/Alb may require further investigation. Second, the number of cases in the present study might have been too small to confirm the robustness of NLR/Alb as a useful prognostic factor. Hence, multicenter prospective studies using validation cohorts are required to validate preoperative NLR/Alb as a prognostic marker in patients with GC after curative surgery.

## 5. Conclusions

In conclusion, preoperative NLR/Alb might be a useful prognostic factor for patients with GC after curative surgery. The evaluation of preoperative NLR/Alb may help select patients who are candidates for aggressive nutritional intervention and perioperative chemotherapy.

## Figures and Tables

**Table 1:** Association of the clinicopathological data between the low- and high-NLR/Alb groups.

Variables	All Patients (*n* = 483)	NLR/Alb		*p*-Value
		Low (*n* = 139)	High (*n* = 344)	
Age (years)	<65	60 (43.2)	114 (33.1)	0.05
	≥65	79 (56.8)	230 (66.9)	
Sex	Male	103 (74.1)	217 (63.1)	0.03
	Female	36 (25.9)	127 (36.9)	
BMI (kg/m^2^)	<18.5	9 (6.5)	40 (11.6)	0.17
	≥18.5, <25.0	94 (67.6)	231 (67.2)	
	≥25	36 (25.9)	73 (21.2)	
Preoperative CRP level	(Mean SD)	0.12 (0.19)	0.22 (0.44)	0.01
Operation	Not TG	109 (78.4)	253 (73.5)	0.30
	TG	30 (21.6)	91 (26.5)	
Tumor size (mm)	≤30	78 (56.1)	169 (49.1)	0.19
	>30	61 (43.9)	175 (50.9)	
Histological type	Well/moderately	74 (53.2)	175 (50.9)	0.69
	Poorly	65 (46.8)	169 (49.1)	
Lymphatic invasion	−	101 (72.7)	232 (67.4)	0.28
	+	38 (27.3)	112 (32.6)	
Venous invasion	−	83 (59.7)	197 (57.3)	0.68
	+	56 (40.3)	147 (42.7)	
pStage	I	100 (71.9)	231 (67.2)	0.33
	II/III	39 (28.1)	113 (32.8)	
Surgical complications	−	116 (83.5)	290 (84.3)	0.89
	+	23 (16.5)	54 (15.7)	
Hospitalization	(Mean SD)	9.81 (7.48)	10.03 (8.94)	0.80

BMI, body mass index; SD, standard deviation; TG, total gastrectomy; PNI, prognostic nutritional index; NLR, neutrophil-to-lymphocyte ratio; CRP, C-reactive protein; pStage, pathological stage; Alb, serum albumin.

**Table 2:** Univariate and multivariate analyses of the clinicopathological factors and preoperative NLR/Alb for overall survival.

Factors		Univariate		*p*-Value	Multivariate		*p*-Value
		HR	95% CI		HR	95% CI	
Age (years)	<65	1			1		
	≥65	2.99	1.33–6.73	0.01	1.98	0.87–4.49	0.10
Sex	Male	1					
	Female	0.66	0.33–1.32	0.24			
BMI (kg/m^2^)	<18.5	1					
	≥18.5, <25.0	0.60	0.26–1.37	0.22			
	≥25	0.42	0.15–1.21	0.11			
Operation	Not TG	1			1		
	TG	2.52	1.38–4.60	0.003	1.37	0.74–2.54	0.32
Tumor size (mm)	≤30	1					
	>30	1.80	0.97–3.33	0.06			
Histological type	Well/moderately	1					
	Poorly	1.50	0.82–2.75	0.19			
Lymphatic invasion	−	1			1		
	+	3.21	1.75–5.87	<0.001	1.02	0.52–2.02	0.95
Venous invasion	−	1			1		
	+	7.62	3.39–17.13	<0.001	3.81	1.50–9.68	0.005
pStage	I	1			1		
	II/III	6.09	3.13–11.87	<0.001	2.65	1.21–5.81	0.02
Surgical complications	−	1					
	+	0.59	0.21–1.67	0.32			
Preoperative CRP	Low	1			1		
	High	1.71	1.01–2.89	0.045	1.38	0.74–2.55	0.31
Preoperative NLR/Alb	Low	1			1		
	High	5.58	1.73–18.05	0.004	4.13	1.26–13.55	0.02

HR, hazard ratio; CI, confidence interval; BMI, body mass index; TG, total gastrectomy; pStage, pathological stage; CRP, C-reactive protein; NLR, neutrophil-to-lymphocyte ratio.

**Table 3:** Univariate and multivariate analyses of the clinicopathological factors and preoperative NLR/Alb for relapse-free survival.

Factors		Univariate		*p*-Value	Multivariate		*p*-Value
		HR	95% CI		HR	95% CI	
Age (years)	<65	1			1		
	≥65	2.12	1.15–3.91	0.02	1.45	0.77–2.72	0.25
Sex	Male	1					
	Female	1.05	0.62–1.79	0.85			
BMI (kg/m^2^)	<18.5	1					
	≥18.5, <25.0	0.51	0.25–1.02	0.29			
	≥25	0.58	0.26–1.30	0.18			
Operation	Not TG	1			1		
	TG	1.97	1.17–3.32	0.01	1.19	0.69–2.05	0.53
Tumor size (mm)	≤30				1		
	≥30	2.34	1.36–4.03	0.002	0.87	0.47–1.60	0.65
Histological type	Well/moderately	1					
	Poorly	1.63	0.97–2.74	0.06			
Lymphatic invasion	−	1			1		
	+	4.56	2.68–7.75	<0.001	2.13	1.03–4.42	0.04
Venous invasion	−	1			1		
	+	5.51	2.98–10.18	<0.001	1.79	0.98–3.25	0.06
pStage	I	1			1		
	II/III	6.87	3.88–12.19	<0.001	3.48	1.68–7.20	<0.001
Surgical complications	−	1					
	+	1.20	0.62–2.30	0.59			
Preoperative CRP	Low	1					
	High	1.66	0.98–2.81	0.06			
Preoperative NLR/Alb	Low	1			1		
	High	3.81	1.64–8.86	0.002	3.16	1.34–7.45	0.009

HR, hazard ratio; CI, confidence interval; BMI, body mass index; TG, total gastrectomy; pStage, pathological stage; CRP, C-reactive protein; NLR, neutrophil-to-lymphocyte ratio; Alb, serum albumin.

## Data Availability

The data presented in this study are available upon request from the corresponding author.

## Supplementary Materials

The following supporting information can be downloaded at: https://www.mdpi.com/article/10.3390/jpm13030432/s1, Figure S1. Definition of the cutoff value by receiver operating characteristic curve analysis on survival in prognostic nutritional index, neutrophil-to-lymphocyte ratio (NLR), and NLR/albumin (NLR/Alb). AUC, area under the curve; PNI, prognostic nutritional index. Figure S2. Subgroup survival analysis according to NLR/Alb by pStage. OS, overall survival, RFS, relapse-free survival. Table S1. Association of the clinicopathological data between the low and high PNI and NLR groups. Table S2. Univariate and multivariate analyses of the clinicopathological factors and preoperative prognostic nutritional index for overall survival. Table S3. Univariate and multivariate analyses of the clinicopathological factors and preoperative prognostic nutritional index for relapse-free survival.

## References

[1] Sung H., Ferlay J., Siegel R.L., Laversanne M., Soerjomataram I., Jemal A., Bray F. (2021). Global Cancer Statistics 2020: GLOBOCAN estimates of incidence and mortality worldwide for 36 cancers in 185 countries. CA Cancer J. Clin..

[2] Smyth E.C., Nilsson M., Grabsch H.I., van Grieken N.C., Lordick F. (2020). Gastric cancer. Lancet.

[3] Sasako M., Sakuramoto S., Katai H., Kinoshita T., Furukawa H., Yamaguchi T., Nashimoto A., Fujii M., Nakajima T., Ohashi Y. (2011). Five-year outcomes of a randomized phase III trial comparing adjuvant chemotherapy with S-1 versus surgery alone in Stage II or III gastric cancer. J. Clin. Oncol..

[4] Yoshida K., Kodera Y., Kochi M., Ichikawa W., Kakeji Y., Sano T., Nagao N., Takahashi M., Takagane A., Watanabe T. (2019). Addition of docetaxel to oral fluoropyrimidine improves efficacy in patients with Stage III gastric cancer: Interim analysis of JACCRO GC-07, a randomized controlled trial. J. Clin. Oncol..

[5] Kang Y.K., Boku N., Satoh T., Ryu M.H., Chao Y., Kato K., Chung H.C., Chen J.S., Muro K., Kang W.K. (2017). Nivolumab in patients with advanced gastric or gastro-oesophageal junction cancer refractory to, or intolerant of, at least two previous chemotherapy regimens (ONO-4538-12, ATTRACTION-2): A randomised, double-blind, placebo-controlled, Phase 3 trial. Lancet.

[6] Janjigian Y.Y., Shitara K., Moehler M., Garrido M., Salman P., Shen L., Wyrwicz L., Yamaguchi K., Skoczylas T., Campos Bragagnoli A. (2021). First-line nivolumab plus chemotherapy versus chemotherapy alone for advanced gastric, gastro-oesophageal junction, and oesophageal adenocarcinoma (CheckMate 649): A randomised, open-label, Phase 3 trial. Lancet.

[7] Shitara K., Özgüroğlu M., Bang Y.-J., Di Bartolomeo M., Mandalà M., Ryu M.-H., Fornaro L., Olesiński T., Caglevic C., Chung H.C. (2018). Pembrolizumab versus paclitaxel for previously treated, advanced gastric or gastro-oesophageal junction cancer (KEYNOTE-061): A randomised, open-label, controlled, Phase 3 trial. Lancet.

[8] Shitara K., Bang Y.J., Iwasa S., Sugimoto N., Ryu M.H., Sakai D., Chung H.C., Kawakami H., Yabusaki H., Lee J. (2020). Trastuzumab deruxtecan in previously treated HER2-positive gastric cancer. N. Engl. J. Med..

[9] Shitara K., Doi T., Dvorkin M., Mansoor W., Arkenau H.-T., Prokharau A., Alsina M., Ghidini M., Faustino C., Gorbunova V. (2018). Trifluridine/tipiracil versus placebo in patients with heavily pretreated metastatic gastric cancer (TAGS): A randomised, double-blind, placebo-controlled, Phase 3 trial. Lancet Oncol..

[10] Zhou C., Hu J., Wang Y., Ji M.H., Tong J., Yang J.J., Xia H. (2021). A machine learning-based predictor for the identification of the recurrence of patients with gastric cancer after operation. Sci. Rep..

[11] Liu X., Lei S., Wei Q., Wang Y., Liang H., Chen L. (2022). Machine learning-based correlation study between perioperative immunonutritional index and postoperative anastomotic leakage in patients with gastric cancer. Int. J. Med. Sci..

[12] Zhang L.X., Wei Z.J., Xu A.M., Zang J.H. (2018). Can the neutrophil-lymphocyte ratio and platelet-lymphocyte ratio be beneficial in predicting lymph node metastasis and promising prognostic markers of gastric cancer patients? tumor maker retrospective study. Int. J. Surg..

[13] Li Z., Li S., Ying X., Zhang L., Shan F., Jia Y., Ji J. (2020). The clinical value and usage of inflammatory and nutritional markers in survival prediction for gastric cancer patients with neoadjuvant chemotherapy and D2 lymphadenectomy. Gastric Cancer.

[14] Hirahara N., Matsubara T., Fujii Y., Kaji S., Kawabata Y., Hyakudomi R., Yamamoto T., Taniura T., Tajima Y. (2020). Comparison of the prognostic value of immunoinflammation-based biomarkers in patients with gastric cancer. Oncotarget.

[15] Wang S.H., Zhai S.T., Lin H. (2016). Role of prognostic nutritional index in patients with gastric cancer: A meta-analysis. Minerva Med..

[16] Takechi H., Fujikuni N., Tanabe K., Hattori M., Amano H., Noriyuki T., Nakahara M. (2020). Using the preoperative prognostic nutritional index as a predictive factor for non-cancer-related death in post-curative resection gastric cancer patients: A retrospective cohort study. BMC Gastroenterol..

[17] Xishan Z., Ye Z., Feiyan M., Liang X., Shikai W. (2020). The role of prognostic nutritional index for clinical outcomes of gastric cancer after total gastrectomy. Sci. Rep..

[18] Zhao Q., Chen S., Feng J.-F. (2017). A Novel inflammation-based prognostic index for patients with esophageal squamous cell carcinoma: Neutrophil lymphocyte ratio/albumin ratio. Oncotarget.

[19] Lv Y., Zhang J., Liu Z., Tian Y., Liu F. (2019). A novel inflammation-based prognostic index for patients with esophageal squamous cell carcinoma: Neutrophil lymphocyte ratio/prealbumin ratio. Medicine.

[20] Ueda K., Ogasawara N., Yonekura S., Matsunaga Y., Hoshino R., Kurose H., Chikui K., Uemura K., Nakiri M., Nishihara K. (2020). The prognostic value of systemic inflammatory markers in advanced renal cell carcinoma patients treated with molecular targeted therapies. Anticancer Res..

[21] Greiner M., Pfeiffer D., Smith R.D. (2000). Principles and practical application of the receiver-operating characteristic analysis for diagnostic tests. Prev. Vet. Med..

[22] Proctor M.J., McMillan D.C., Morrison D.S., Fletcher C.D., Horgan P.G., Clarke S.J. (2012). A derived neutrophil to lymphocyte ratio predicts survival in patients with cancer. Br. J. Cancer.

[23] Maiorino L., Daßler-Plenker J., Sun L., Egeblad M. (2022). Innate immunity and cancer pathophysiology. Annu. Rev. Pathol..

[24] Arends J., Bachmann P., Baracos V., Barthelemy N., Bertz H., Bozzetti F., Fearon K., Hütterer E., Isenring E., Kaasa S. (2017). ESPEN Guidelines on nutrition in cancer patients. Clin. Nutr..

[25] Kanda M., Mizuno A., Tanaka C., Kobayashi D., Fujiwara M., Iwata N., Hayashi M., Yamada S., Nakayama G., Fujii T. (2016). Nutritional predictors for postoperative short-term and long-term outcomes of patients with gastric cancer. Medicine.

[26] Lee J.Y., Kim H.-I., Kim Y.-N., Hong J.H., Alshomimi S., An J.Y., Cheong J.-H., Hyung W.J., Noh S.H., Kim C.-B. (2016). Clinical significance of the prognostic nutritional index for predicting short- and long-term surgical outcomes after gastrectomy: A retrospective analysis of 7781 gastric cancer patients. Medicine.

[27] Shimada H., Takiguchi N., Kainuma O., Soda H., Ikeda A., Cho A., Miyazaki A., Gunji H., Yamamoto H., Nagata M. (2010). High preoperative neutrophil-lymphocyte ratio predicts poor survival in patients with gastric cancer. Gastric Cancer.

[28] Wang S.C., Chou J.F., Strong V.E., Brennan M.F., Capanu M., Coit D.G. (2016). Pretreatment neutrophil to lymphocyte ratio independently predicts disease-specific survival in resectable gastroesophageal junction and gastric adenocarcinoma. Ann. Surg..

[29] Xin-Ji Z., Yong-Gang L., Xiao-Jun S., Xiao-Wu C., Dong Z., Da-Jian Z. (2015). The prognostic role of neutrophils to lymphocytes ratio and platelet count in gastric cancer: A meta-analysis. Int. J. Surg..

[30] Jiang N., Deng J.Y., Ding X.W., Ke B., Liu N., Zhang R.P., Liang H. (2014). Prognostic nutritional index predicts postoperative complications and long-term outcomes of gastric cancer. World J. Gastroenterol..

[31] Aoyama T., Nakazono M., Segami K., Nagasawa S., Kano K., Yamada T., Maezawa Y., Hara K., Hashimoto I., Suematsu H. (2021). The clinical influence of the C-reactive protein-to-albumin ratio in patients who received curative treatment for gastric cancer. In Vivo.

[32] Xu B.B., Lu J., Zheng Z.F., Xie J.W., Wang J.B., Lin J.X., Chen Q.Y., Cao L.L., Lin M., Tu R.H. (2019). The predictive value of the preoperative C-reactive protein-albumin ratio for early recurrence and chemotherapy benefit in patients with gastric cancer after radical gastrectomy: Using randomized phase III trial data. Gastric Cancer.

[33] Okugawa Y., Toiyama Y., Yamamoto A., Shigemori T., Ichikawa T., Yin C., Suzuki A., Fujikawa H., Yasuda H., Hiro J. (2020). Lymphocyte-to-C-reactive protein ratio and score are clinically feasible nutrition-inflammation markers of outcome in patients with gastric cancer. Clin. Nutr..

[34] Xiong J., Hu H., Kang W., Liu H., Ma F., Ma S., Li Y., Jin P., Tian Y. (2021). Prognostic impact of preoperative naples prognostic score in gastric cancer patients undergoing surgery. Front. Surg..

[35] Ongaro E., Buoro V., Cinausero M., Caccialanza R., Turri A., Fanotto V., Basile D., Vitale M.G., Ermacora P., Cardellino G.G. (2017). Sarcopenia in gastric cancer: When the loss costs too much. Gastric Cancer.

[36] Qian S., Golubnitschaja O., Zhan X. (2019). Chronic inflammation: Key player and biomarker-set to predict and prevent cancer development and progression based on individualized patient profiles. EPMA J..

[37] Zavros Y., Merchant J.L. (2022). The immune microenvironment in gastric adenocarcinoma. Nat. Rev. Gastroenterol. Hepatol..

[38] Liotti F., Marotta M., Melillo R.M., Prevete N. (2022). The impact of resolution of inflammation on tumor microenvironment: Exploring new ways to control cancer progression. Cancers.

[39] Zhao J., Jin J. (2022). Neutrophil extracellular traps: New players in cancer research. Front. Immunol..

[40] Li T.J., Jiang Y.M., Hu Y.F., Huang L., Yu J., Zhao L.Y., Deng H.J., Mou T.Y., Liu H., Yang Y. (2017). Interleukin-17-producing neutrophils link inflammatory stimuli to disease progression by promoting angiogenesis in gastric cancer. Clin. Cancer Res..

[41] Zhang W., Gu J., Chen J., Zhang P., Ji R., Qian H., Xu W., Zhang X. (2017). Interaction with neutrophils promotes gastric cancer cell migration and invasion by inducing epithelial-mesenchymal transition. Oncol. Rep..

[42] Wang T.T., Zhao Y.L., Peng L.S., Chen N., Chen W., Lv Y.P., Mao F.Y., Zhang J.Y., Cheng P., Teng Y.S. (2017). Tumour-activated neutrophils in gastric cancer foster immune suppression and disease progression through GM-CSF-PD-L1 pathway. Gut.

[43] Hiramatsu S., Tanaka H., Nishimura J., Yamakoshi Y., Sakimura C., Tamura T., Toyokawa T., Muguruma K., Yashiro M., Hirakawa K. (2020). Gastric cancer cells alter the immunosuppressive function of neutrophils. Oncol. Rep..

[44] Lim Y.J., Koh J., Kim K., Chie E.K., Kim B., Lee K.B., Jang J.-Y., Kim S.-W., Oh D.-Y., Bang Y.-J. (2015). High ratio of programmed cell death protein 1 (PD-1)(+)/CD8(+) tumor-infiltrating lymphocytes identifies a poor prognostic subset of extrahepatic bile duct cancer undergoing surgery plus adjuvant chemoradiotherapy. Radiother. Oncol..

[45] Miura T., Yoshizawa T., Hirai H., Seino H., Morohashi S., Wu Y., Wakiya T., Kimura N., Kudo D., Ishido K. (2017). Prognostic impact of CD163+ macrophages in tumor stroma and CD8+ T-cells in cancer cell nests in invasive extrahepatic bile duct cancer. Anticancer Res..

[46] Djenidi F., Adam J., Goubar A., Durgeau A., Meurice G., de Montpréville V., Validire P., Besse B., Mami-Chouaib F. (2015). CD8+CD103+ tumor-infiltrating lymphocytes are tumor-specific tissue-resident memory T cells and a prognostic factor for survival in lung cancer patients. J. Immunol..

[47] Xu J., Guo R., Jia J., He Y., He S. (2021). Activation of toll-like receptor 2 enhances peripheral and tumor-infiltrating CD8+ T cell cytotoxicity in patients with gastric cancer. BMC Immunol..

[48] Feng F., Zheng G., Wang Q., Liu S., Liu Z., Xu G., Wang F., Guo M., Lian X., Zhang H. (2018). Low lymphocyte count and high monocyte count predicts poor prognosis of gastric cancer. BMC Gastroenterol..

[49] Yeun J.Y., Kaysen G.A. (1998). Factors influencing serum albumin in dialysis patients. Am. J. Kidney Dis..

[50] Oñate-Ocaña L.F., Aiello-Crocifoglio V., Gallardo-Rincón D., Herrera-Goepfert R., Brom-Valladares R., Carrillo J.F., Cervera E., Mohar-Betancourt A. (2007). Serum albumin as a significant prognostic factor for patients with gastric carcinoma. Ann. Surg. Oncol..

[51] Mulazzani G.E.G., Corti F., Della Valle S., Di Bartolomeo M. (2021). Nutritional support indications in gastroesophageal cancer patients: From perioperative to palliative systemic therapy. A comprehensive review of the last decade. Nutrients.

[52] Tsuburaya A., Mizusawa J., Tanaka Y., Fukushima N., Nashimoto A., Sasako M., Stomach Cancer Study Group of the Japan Clinical Oncology Group (2014). Neoadjuvant chemotherapy with S-1 and cisplatin followed by D2 gastrectomy with para-aortic lymph node dissection for gastric cancer with extensive lymph node metastasis. Br. J. Surg..

[53] Iwasaki Y., Terashima M., Mizusawa J., Katayama H., Nakamura K., Katai H., Yoshikawa T., Ito S., Kaji M., Kimura Y. (2021). Gastrectomy with or without neoadjuvant S-1 plus cisplatin for type 4 or large type 3 gastric cancer (JCOG0501): An open-label, phase 3, randomized controlled trial. Gastric Cancer.

