# Supplementary material for: Clinical Effects of the Neutrophil-to-Lymphocyte Ratio/Serum Albumin Ratio in Patients with Gastric Cancer after Gastrectomy
Source: J Pers Med. 2023 Feb 28;13(3):432. doi: 10.3390/jpm13030432 (PMC10051294; doi:10.3390/jpm13030432)
Supplement: Supplementary file 1 [file jpm-13-00432-s001.zip › Supplementary_Figure.pptx]

## Slide 1
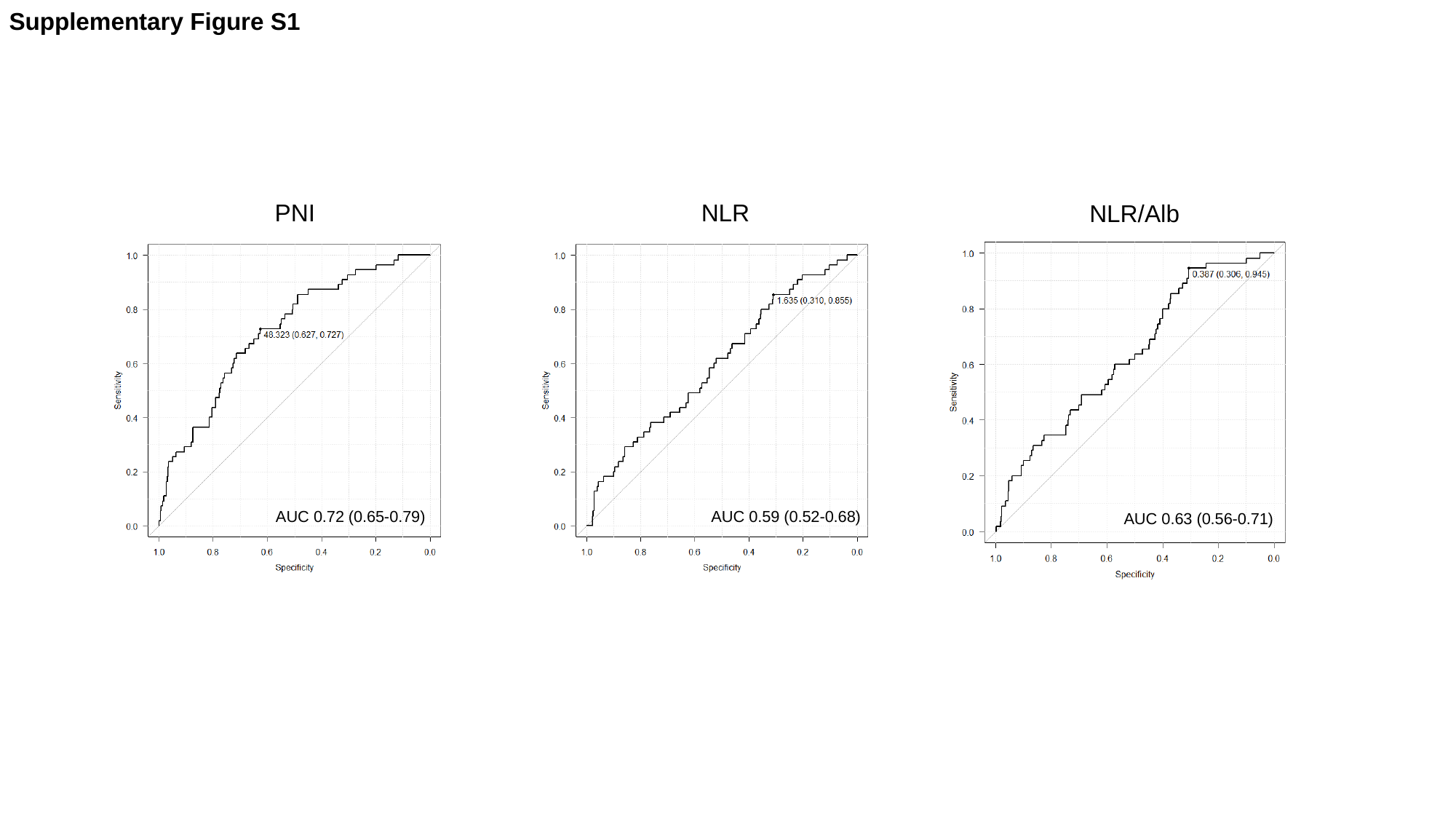

Supplementary Figure S1
PNI
NLR
NLR/Alb
AUC 0.72 (0.65-0.79)
AUC 0.59 (0.52-0.68)
AUC 0.63 (0.56-0.71)

## Slide 2
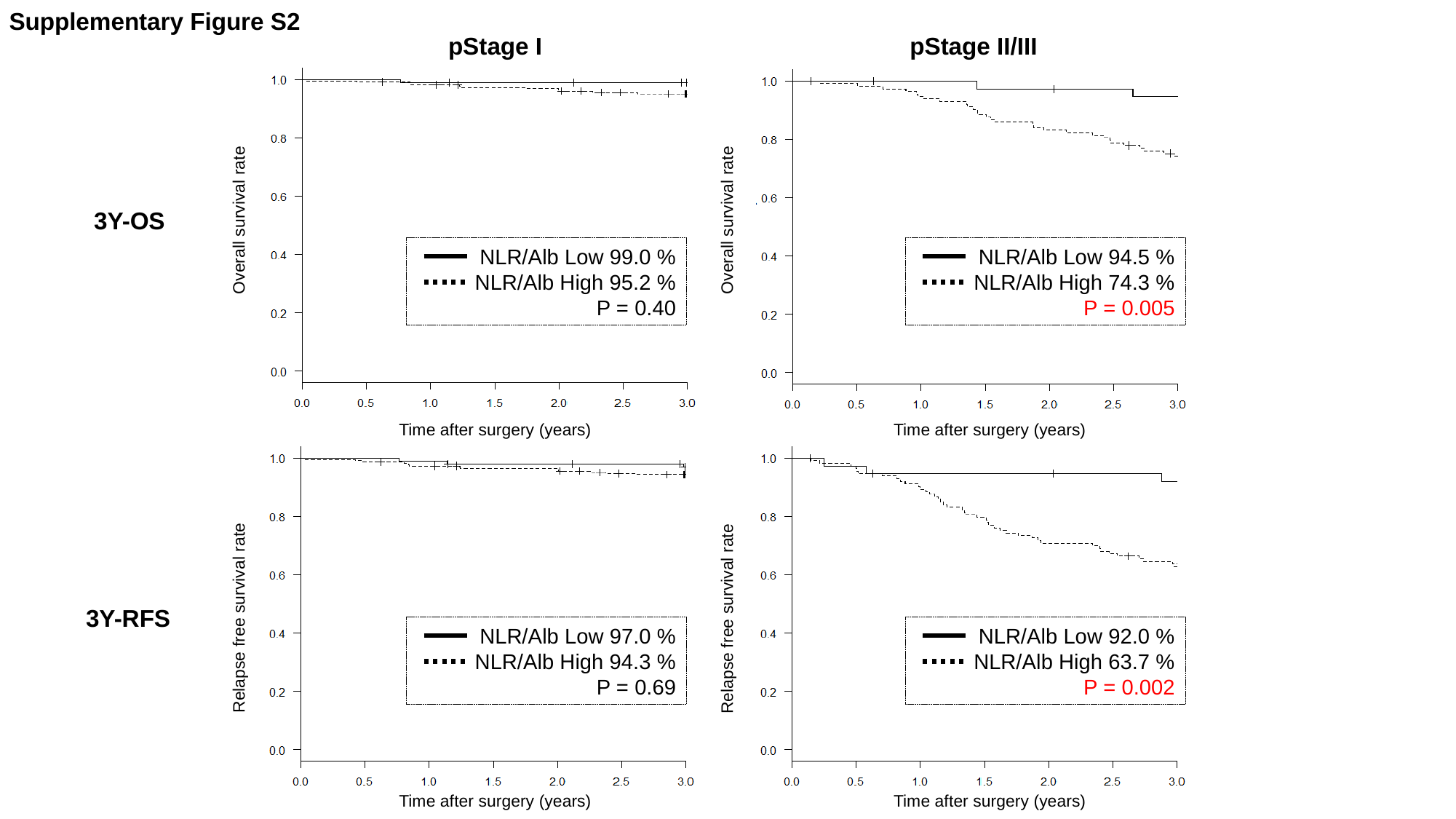

Supplementary Figure S2
pStage I
pStage II/III
3Y-OS
Overall survival rate
Overall survival rate
NLR/Alb Low 99.0 %
NLR/Alb High 95.2 %
P = 0.40
NLR/Alb Low 94.5 %
NLR/Alb High 74.3 %
P = 0.005
Time after surgery (years)
Time after surgery (years)
3Y-RFS
Relapse free survival rate
Relapse free survival rate
NLR/Alb Low 97.0 %
NLR/Alb High 94.3 %
P = 0.69
NLR/Alb Low 92.0 %
NLR/Alb High 63.7 %
P = 0.002
Time after surgery (years)
Time after surgery (years)
