# Supplementary material for: Clinical Effects of the Neutrophil-to-Lymphocyte Ratio/Serum Albumin Ratio in Patients with Gastric Cancer after Gastrectomy
Source: J Pers Med. 2023 Feb 28;13(3):432. doi: 10.3390/jpm13030432 (PMC10051294; doi:10.3390/jpm13030432)
Supplement: Supplementary file 1 [file jpm-13-00432-s001.zip › Supplementary_Table_S1-3_.pdf]

Table S1. Association of the clinicopathological data between the low and high PNI and NLR group.

Variables	All patients (n = 483)	PNI		*P*-value	NLR		*P*-value
		Low (n = 191)	High (n = 292)		Low (n = 144)	High (n = 339)	
Age	< 65	45 (23.6)	129 (44.2)	**< 0.001**	57 (39.6)	117 (34.5)	0.30
	≥ 65	146 (76.4)	163 (55.8)		87 (60.4)	222 (65.5)	
Sex	Male	115 (60.2)	205 (70.2)	**0.02**	97 (67.4)	223 (65.8)	0.75
	Female	76 (39.8)	87 (29.8)		47 (32.6)	116 (34.2)	
BMI	< 18.5	34 (17.8)	15 (5.1)	**< 0.001**	12 (8.3)	37 (10.9)	0.70
	≥ 18.5 < 25.0	125 (65.4)	200 (68.5)		98 (68.1)	227 (67.0)	
	≥ 25	32 (16.8)	77 (26.4)		34 (23.6)	75 (22.1)	
Preoperative CRP level	(Mean SD)	0.28 (0.52)	0.14 (0.26)	**< 0.001**	0.13 (0.20)	0.22 (0.44)	0.02
Operation	Not TG	131 (68.6)	231 (79.1)	**0.01**	114 (79.2)	248 (73.2)	0.17
	TG	60 (31.4)	61 (20.9)		30 (20.8)	91 (26.8)	
Tumor size	≤ 30	70 (36.6)	177 (60.6)	**< 0.001**	78 (54.2)	169 (49.9)	0.43
	> 30	121 (63.4)	115 (39.4)		66 (45.8)	170 (50.1)	
Histological type	Well / moderately	101 (52.9)	148 (50.7)	0.64	75 (52.1)	174 (51.3)	0.92
	Poorly	90 (47.1)	144 (49.3)		69 (47.9)	165 (48.7)	
Lymphatic invasion	–	119 (62.3)	214 (73.3)	**0.01**	104 (72.2)	229 (67.6)	0.34
	+	72 (37.7)	78 (26.7)		40 (27.8)	110 (32.4)	
Venous invasion	–	95 (49.7)	185 (63.4)	**0.003**	89 (61.8)	191 (56.3)	0.27
	+	96 (50.3)	107 (36.6)		55 (38.2)	148 (43.7)	
pStage	I	111 (58.1)	220 (75.3)	**<0.001**	105 (72.9)	226 (66.7)	0.20
	II / III	80 (41.9)	72 (24.7)		39 (27.1)	113 (33.3)	
Surgical complications	–	160 (83.8)	246 (84.2)	0.90	124 (86.1)	282 (83.2)	0.50
	+	31 (16.2)	46 (15.8)		20 (13.9)	57 (16.8)	
Hospitalization	(Mean SD)	10.18 (9.09)	9.83 (8.17)	0.65	9.46 (6.89)	10.18 (9.15)	0.39

BMI, body mass index; SD, standard deviation; TG, total gastrectomy; PNI, prognostic nutrition-al index; NLR, neutrophil-to-lymphocyte ratio; CRP, C-reactive protein; pStage, pathological stage

Table S2. Univariate and multivariate analyses of the clinicopathological factors and preoperative PNI for overall survival

Factors		Univariate		*P*-Value	Multivariate		*P*-Value
		HR	95%CI		HR	95%CI	
Age	< 65	1			1		
	≥ 65	2.99	1.33–6.73	**0.01**	1.99	0.87–4.59	0.11
Sex	Male	1					
	Female	0.66	0.33–1.32	0.24			
BMI	< 18.5	1					
	≥ 18.5, < 25.0	0.60	0.26–1.37	0.22			
	≥ 25	0.42	0.15–1.21	0.11			
Operation	not TG	1			1		
	TG	2.52	1.38–4.60	**0.003**	1.45	0.78–2.69	0.24
Tumor size	≤ 30	1					
	> 30	1.80	0.97–3.33	0.06			
Histological type	Well / Moderately	1					
	Poorly	1.50	0.82–2.75	0.19			
Lymphatic invasion	-	1			1		
	+	3.21	1.75–5.87	**< 0.001**	1.11	0.57–2.19	0.76
Venous invasion	-	1			1		
	+	7.62	3.39–17.13	**< 0.001**	3.67	1.44–9.36	**0.007**
pStage	I	1			1		
	II / III	6.09	3.13–11.87	**< 0.001**	2.35	1.05–5.28	**0.04**
Surgical complications	-	1					
	+	0.59	0.21–1.67	0.32			
Preoperative CRP	Low	1			1		
	High	1.71	1.01-2.89	**0.045**	1.33	0.71-2.48	0.37
Preoperative PNI	High	1			1		
	Low	3.36	1.77–6.35	**< 0.001**	1.82	0.92–3.57	0.09

HR, Hazard Ratio; CI, Confidence interval; BMI, body mass index; TG, total gastrectomy; pStage, pathological Stage; CRP, C-reactive protein; PNI, Prognostic nutritional index

Table S3. Univariate and multivariate analyses of the clinicopathological factors and preoperative PNI for relapse free survival.

Factors		Univariate		P-Value	Multivariate		P-Value
		HR	95%CI		HR	95%CI	
Age	<65	1					
	≥65	2.12	1.15–3.91	**0.02**	1.57	0.83–2.96	0.17
Sex	Male	1					
	Female	1.05	0.62–1.79	0.85			
BMI	< 18.5	1					
	≥18.5 , < 25.0	0.51	0.25–1.02	0.29			
	≥25	0.58	0.26–1.30	0.18			
Operation	not TG	1			1		
	TG	1.97	1.17–3.32	**0.01**	1.27	0.74–2.17	0.39
Tumor size	≤30				1		
	>30	2.34	1.36–4.03	**0.002**	0.87	0.47–1.61	0.65
Histological type	Well / Moderately	1					
	Poorly	1.63	0.97–2.74	0.06			
Lymphatic invasion	-	1			1		
	+	4.56	2.68–7.75	**< 0.001**	1.86	1.02–3.39	**0.04**
Venous invasion	-	1			1		
	+	5.51	2.98–10.18	**< 0.001**	2.13	1.03–4.41	**0.04**
pStage	I	1			1		
	II / III	6.87	3.88–12.19	**< 0.001**	3.24	1.56–6.71	**0.002**
Surgical complications	-	1					
	+	1.20	0.62–2.30	0.59			
Preoperative CRP	Low	1					
	High	1.66	0.98-2.81	0.06			
preoperative PNI	High	1			1		
	Low	2.27	1.35–3.85	**0.002**	1.37	0.79–2.38	0.26

HR, Hazard Ratio; CI, Confidence interval; BMI, body mass index; TG, total gastrectomy; pStage, pathological Stage; CRP, C-reactive protein; PNI, Prognostic nutritional index
